# Bart syndrome with musculoskeletal deformity: a rare case report

**DOI:** 10.1097/MS9.0000000000002732

**Published:** 2024-11-13

**Authors:** Sanish Pokhrel, Zenish Niraula, Pradip Ghimire, Sugam Ale Magar, Ashish Acharya, Kiran Awal

**Affiliations:** Nepal Medical College and Teaching Hospital, Kathmandu, Nepal

**Keywords:** aplasia cutis congenita, Bart syndrome, case report, epidermolysis bullosa, musculoskeletal deformity

## Abstract

**Introduction:**

Bart syndrome is a rare genetic disorder characterized by epidermolysis bullosa (EB), aplasia cutis congenita, that is congenital local absence of skin and nail abnormalities.

**Case Presentation:**

The authors herein, present a case of a 14-year-old boy with Bart syndrome. The syndrome was diagnosed clinically. On examination, multiple generalized blisters were present and absence of nails in the toes of both feet and the middle finger of the left hand, which was associated with musculoskeletal deformity.

**Discussion:**

Bart syndrome, an inherited autosomal dominant disorder, is an exceedingly rare disorder. Musculoskeletal deformity is an uncommon presentation of this syndrome. It is mostly associated with Dystrophic type of EB. It is mostly a clinical diagnosis; however, histopathological study, direct immunofluorescence, and genetic testing helps in diagnosing the type of EB.

**Conclusion:**

The absence of skin in a localized area at birth is a crucial indicator for diagnosing Bart syndrome at birth, which later heals and can obscure the diagnosis. Early diagnosis and conservative management prevent the disease progression and complications.

## Introduction

Highlights

- Bart syndrome is a rare congenital syndrome mostly consisting of congenital local absence of skin, epidermolysis bullosa, and nail abnormalities.
- It can be diagnosed clinically; however, histopathology and immunofluorescence test help to delineate epidermolysis bullosa.
- Any musculoskeletal deformity associated with Bart syndrome is a rare occurrence.

Bart syndrome is a rare genetic disorder characterized by epidermolysis bullosa (EB), aplasia cutis congenital, that is congenital local absence of skin and nail abnormalities^1^. Among the subtypes of epidermolysis bullosa, that is simplex, junctional, or dystrophic, dystrophic EB is more common^2^. The lesions are generally unilateral and mostly involve the medial or dorsal surface of the limbs. The lesions on the limbs are sharply demarcated, glistening red ulcerations that extend upward from the dorsal and medial surface of the foot to the shin^1^. It has been hypothesized that these skin defects can lead to scar formation and contractures, which ultimately cause musculoskeletal deformities, especially in young patients^3^.

We herein present an interesting case of Bart syndrome with musculoskeletal deformity in a 14-year-old boy. Our case had deformities since birth so deformity caused due to contracture could be ruled out, which is unique for such a case of Bart syndrome. This is the first rare case report of Bart syndrome with musculoskeletal deformity from Nepal. This case report has been reported in accordance of 2020 Surgical CAse Report (SCARE) guidelines^4^.

## Case presentation

**Figure 1:**
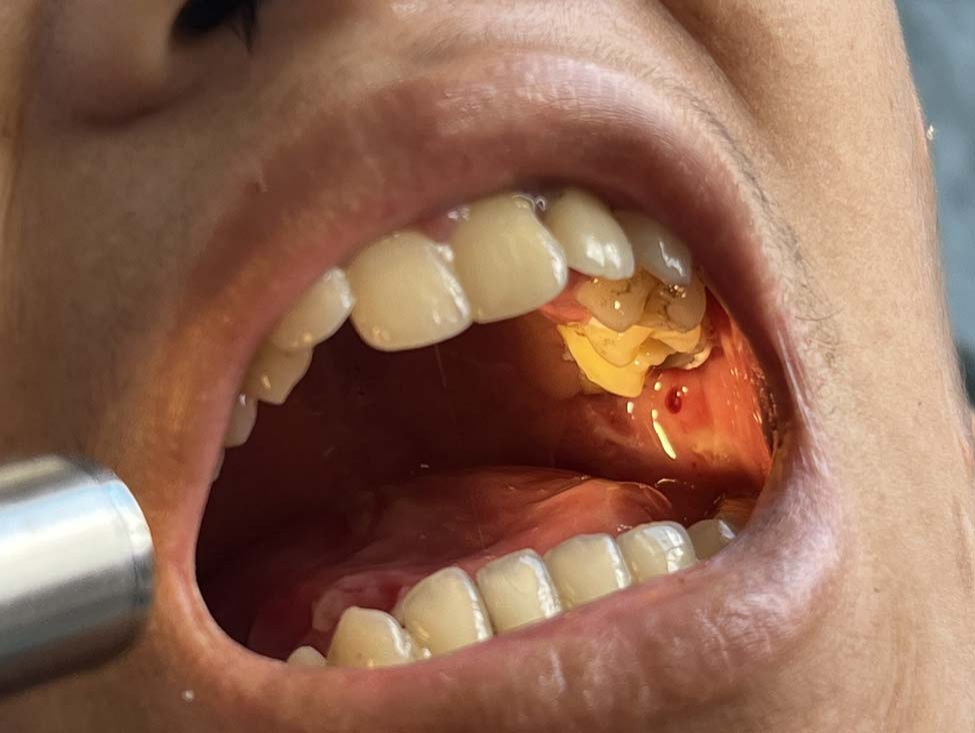
Erosive lesions in the dorsal surface of the tongue, buccal mucosa, and enamel hypoplasia.

**Figure 2:**
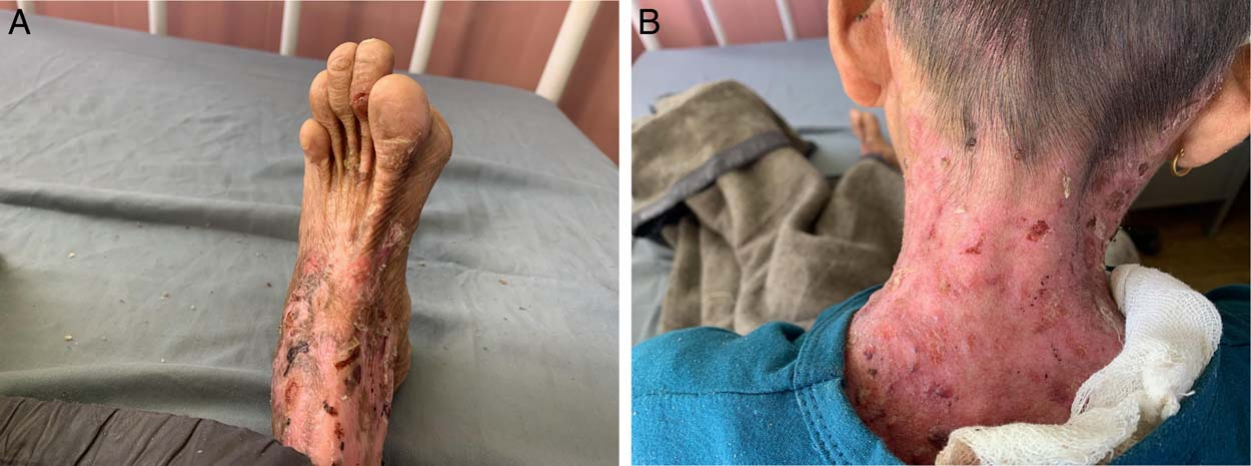
(A) Superficial erosions, scarring, and contracture with absence of nails in left toes; (B) Blisters and granulation tissue in the posterior region of the neck.

A 14-year-old boy, the only child of nonconsanguineous healthy parents, from Rural Nepal presented to the otorhinolaryngology OPD of a tertiary health center with chief complaints of throat pain and dysphagia for one day. The throat pain was intermittent for a few hours. A similar throat pain history was present once or twice a year. He had dysphagia with drooling of saliva and a history of recurrent blood-mixed saliva once or twice a year. On examination of the throat, there was congestion of the posterior pharyngeal wall and erosive lesions in the dorsal surface of the tongue and buccal mucosa (Fig. 1). The patient had tongue-tie and opening the mouth was restricted. Fibrotic bands were also seen in the palatoglossal arch and buccal mucosa inside of the cheek. Enamel hypoplasia was present in Figure 1. Generalized blisters were present mostly in the neck, abdominal area, and flexor aspects of both limbs. The affected part appeared red, devoid of skin with superficial erosions, and scarring. Granulation tissue was present in the posterior region of the neck (Fig. 2). Absence of nails were seen in the toes of both feet and the middle finger of the left hand (Fig. 2).

**Figure 3:**
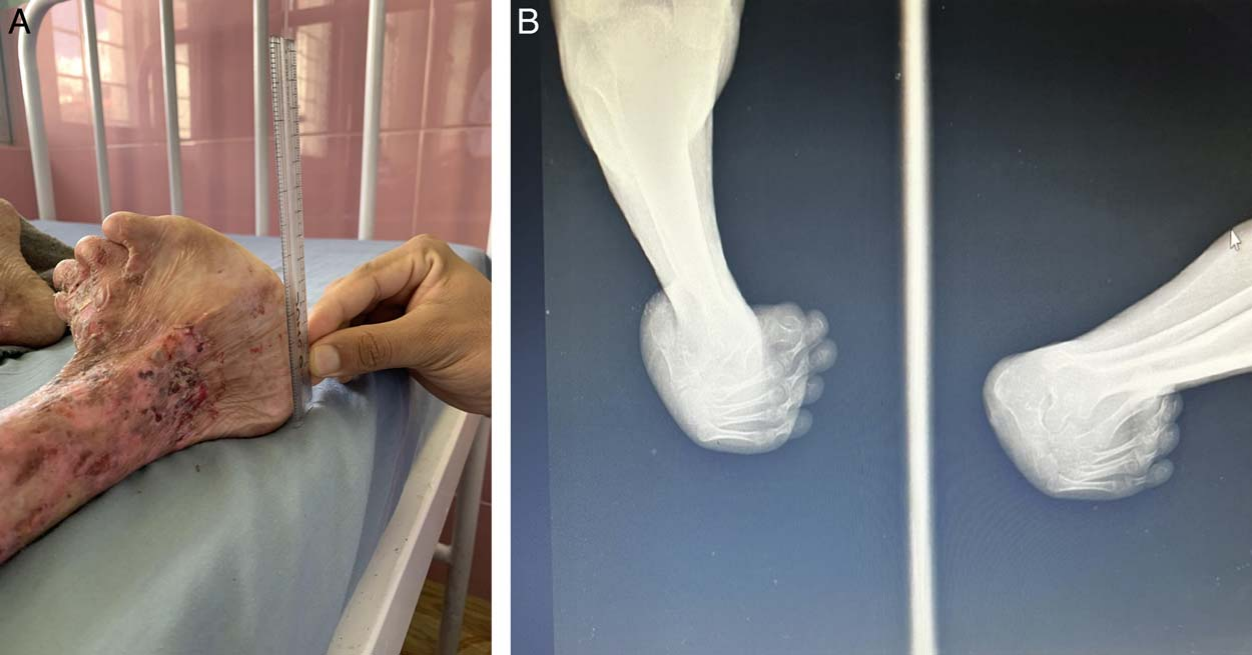
(A) Absence of nails with right limb shortening, adductus, and varus deformity; (B) X-ray showing right limb adductus and varus deformity.

**Figure 4:**
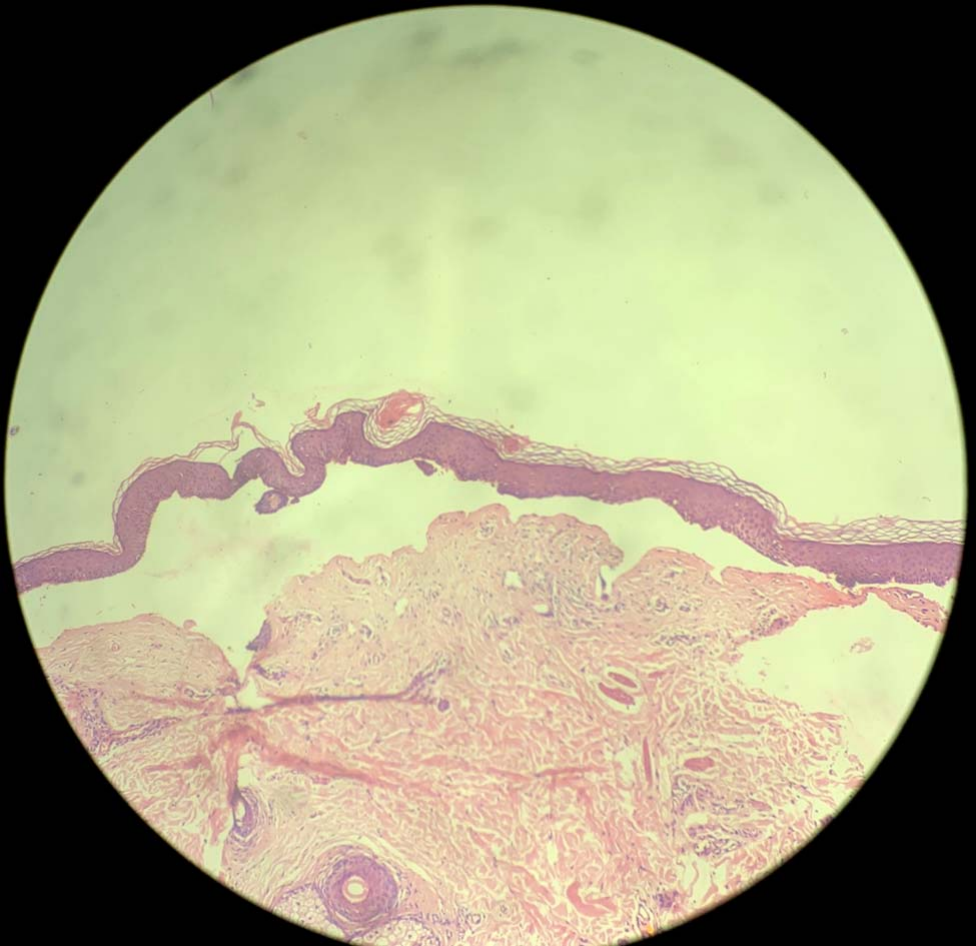
Histopathological finding showing subepidermal blisters with mixed inflammatory cell dermal infiltrate.

There was gross deformity of the right foot with limb shortening, adductus and varus deformity, which was further corroborated by an X-ray of the ankle (Fig. 3). Flexor deformity was also noted in the fingers. There was no similar history present amongst his family members. In a histopathological study of the skin, subepidermal blisters with mixed inflammatory cell dermal infiltrate are seen that are consistent with epidermolysis bullosa (Fig. 4).

The pregnancy was confirmed at the hospital after 2 months of cessation of menstruation. Thereafter, no antenatal visits were done. No folic acid, iron, or calcium tablets were taken by the mother. The mother also gave a history of fever for 2 weeks, in the seventh month of pregnancy, which resolved by itself without any medication. He was delivered at home by normal vaginal delivery, and his parents noticed reddish discoloration and absence of skin in the right leg from mid-tibia to ankle joint and absence of nails, and gross deformity in the right leg immediately after birth. On the second day of birth, he developed a blister at the thumb area and within 10 days there were generalized blisters present. The patient underwent conservative management, including ointment, protective dressings, and antibiotics, after which the congenital absence of skin healed completely; however, the blisters did not subside. The blisters were more present during warm weather and sunlight exposure. Any friction or trauma to the skin also induced blister formation, which did not rupture spontaneously.

All the developmental milestones were achieved normally. He is currently studying with good school performance. He has not received any vaccination since birth mostly due to being from a rural area and frequent hospital visits for this condition.

The patient was managed with betamethasone dipropionate and gentamicin cream, placental extract cream, emollients, and conservative treatment with local care and vaseline gauze dressing. Throat pain subsided after taking oral drops of betamethasone, fexofenadine, and repeated mouth gargle.

## Discussion

Bart syndrome, an exceedingly rare disorder, was named after Bruce J. Bart after describing this syndrome in a large family in 1966. It is an inherited autosomal dominant disorder characterized by congenital absence of skin, blistering, and nail deformity^5^. Genetic analysis demonstrated abnormalities in linkage of inheritance to the region of chromosome 3 near the type VII collagen gene^6^. Bart syndrome is mostly associated with dystrophic type of EB. Genetic mutations in COL7A1 gene, which encodes type VII collagen, a component that secures the basement membrane hemidesmosomes to the dermis, and in utero leg rubbing are considered the etiology for aplasia cutis congenita^7^. Musculoskeletal deformity is an uncommon presentation of Bart syndrome; however, in this case, musculoskeletal deformity is prominent. The rupture of bullous skin lesions can lead to secondary inflammatory and fibrotic changes, which on repetitive episodes can complicate to scarring and musculoskeletal deformities. The repeated episodes of scarring will compromise the blood supply to the distal limbs and bones. This has been reported to cause muscle atrophy and hindrance in the growth of distal bones. The distal phalanx of hands and feet may show shortening or have taper ends^8^. This is also evidenced in the X-ray image of our case report. On inspection, this might give an impression that the deformity is due to fibrotic scar resulting in contracture formation, but in our case, the deformity has been present since birth, so deformity due to contracture can be ruled out.

Bart syndrome is mostly a clinical diagnosis. Histopathological examination, direct immunofluorescence, and genetic testing help in diagnosing the type of epidermolysis bullosa^1^. Localized absence of skin at birth, an important finding for diagnosis of Bart syndrome, is an early finding; however, our case presented in early adolescence so diagnosis was made on the basis of history.

In more severe cases particularly when associated with junctional epidermolysis bullosa, it may be accompanied by other abnormalities most often pyloric atresia, ureteral stenosis, renal abnormalities, arthrogryposis, abnormal ears and nose, and amniotic bands^9^. Management of this syndrome varies from conservative care to a comprehensive approach. Conservative care includes attending to the wound locally and preventing infections. Local wound care includes applying topical treatments like silver sulfadiazine cream or bacitracin ointment and using nonadhesive dressings like petrolatum gauze. For chronic, large nonhealing wounds or those with complications like contracture formation, surgical intervention is necessary. Procedures may involve using skin autografts or allografts^10^.

The prognosis for Bart syndrome varies depending on factors such as the severity of Aplasia Cutis Congenita, the type of epidermolysis bullosa, and how well the treatment works. Generally, the prognosis for this condition is considered to be reasonable^1^.

Finding out localized absence of skin in neonates should prompt one to look for other features suggestive of syndromic association like Bart syndrome and conservative treatment on preventing its complications should be counseled.

## Strength and limitations

This is the first reported case of Bart syndrome with musculoskeletal deformities among the Nepalese population. Despite musculoskeletal deformities being uncommon among the case of Bart syndrome, our case presents this prominently. However, a further investigation and analysis of why such deformities occurred in this case could not be accomplished due to lack of respective resources. Similarly, the past history given by the informant could be biased. These are the limitations of our study. Nevertheless, we hope our literature will add vital information to the existing literatures on Bart syndrome with musculoskeletal deformities.

## Conclusion

Bart syndrome manifests as a rare genetic disorder marked by congenital absence of skin, blistering, and nail deformities. The absence of skin in a localized area at birth is a crucial indicator for diagnosing Bart syndrome at birth which later heals and can obscure the diagnosis. All associated disorders should be thoroughly examined and treated as early as possible for good outcomes. Management of the case depends on the severity but early diagnosis and conservative management prevent the disease progression to severity and avert complex therapeutic or surgical interventions.

## Ethical approval

Case reports are exempt from ethical approval in our institution, Nepal Medical College and Teaching Hospital, Attarkhel, Kathmandu.

## Consent

Written informed consent was obtained from the patient’s parents for publication and any accompanying images. A copy of the written consent is available for review by the Editor-in-Chief of this journal on request.

## Source of funding

Not applicable.

## Author contribution

All authors contributed equally on conceptualization, design, acquisition of patient information, previous literature review, preparation of draft, and editing.

## Conflicts of interest disclosure

The authors declare no conflicts of interest.

## Research registration unique identifying number (UIN)

Not applicable.

## Guarantor

Sanish Pokhrel, Nepal Medical College and Teaching Hospital, Kathmandu University, Attarkhel, Kathmandu, Nepal. Tel.: +977 9843838837. E-mail: sanispokh.45@gmail.com.

## Data availability statement

The datasets generated during the study are publicly available, available upon reasonable request.

## Provenance and peer review

Not commissioned, externally peer-reviewed.
